# Affinity, potency, efficacy, and selectivity of neurokinin A analogs at human recombinant NK2 and NK1 receptors

**DOI:** 10.1371/journal.pone.0205894

**Published:** 2018-10-25

**Authors:** Nadia M. J. Rupniak, Elisabetta Perdona, Cristiana Griffante, Palmina Cavallini, Anna Sava, Daniel J. Ricca, Karl B. Thor, Edward C. Burgard, Mauro Corsi

**Affiliations:** 1 Dignify Therapeutics, Research Triangle Park, North Carolina, United States of America; 2 Drug Design and Discovery, Aptuit an Evotec Company, Verona, Italy; Cinvestav-IPN, MEXICO

## Abstract

A series of peptide NK2 receptor agonists was evaluated for affinity, potency, efficacy, and selectivity at human recombinant NK2 and NK1 receptors expressed in CHO cells to identify compounds with the greatest separation between NK2 and NK1 receptor agonist activity. Binding studies were performed using displacement of [^125^I]-NKA binding to NK2 receptors and displacement of [^3^H]-Septide binding to NK1 receptors expressed in CHO cells. Functional studies examining the increase in intracellular calcium levels and cyclic AMP stimulation were performed using the same cell lines. A correlation was demonstrated between binding affinities (Ki) and potency to increase intracellular calcium (EC_50_) for NK2 and NK1 receptors. Ranking compounds by their relative affinity (Ki) or potency (EC_50_) at NK2 or NK1 receptors indicated that the most selective NK2 agonists tested were [Lys^5^,MeLeu^9^,Nle^10^]-NKA(4-10) (NK1/NK2 Ki ratio = 674; NK1/NK2 EC_50_ ratio = 105) and [Arg^5^,MeLeu^9^,Nle^10^]-NKA(4-10) (NK1/NK2 Ki ratio = 561; NK1/NK2 EC_50_ ratio = 70). The endogenous peptide, NKA, lacked selectivity with an NK1/NK2 Ki ratio = 20 and NK1/NK2 EC_50_ ratio = 1. Of the compounds selected for evaluation in cyclic AMP stimulation assays, [β-Ala^8^]-NKA(4–10) had the greatest selectivity for activation of NK2 over NK1 receptors (NK1/NK2 EC_50_ ratio = 244), followed by [Lys^5^,MeLeu^9^,Nle^10^]-NKA(4-10) (ratio = 74), and NKA exhibited marginal selectivity (ratio = 2.8).

## Introduction

Agonists acting at tachykinin NK2 receptors have potential as a new class of therapeutics to stimulate bladder and rectal voiding ‘on demand’ in patients with impaired voluntary control over urination and defecation. The ability of the endogenous peptide, neurokinin A (NKA), and other NK2 agonists, to contract bladder and colon smooth muscle preparations from various species (including human) has been amply documented [1–5]. Administration of NK2 agonists to anesthetized animals increases bladder tone, contractility, and distension-evoked responses [6–9], and increases gastrointestinal motility [10, 11] and colorectal pressure [8, 9, 12].

The rapid inactivation of NKA and related peptides by protease enzymes in vivo means that on-demand voiding may be completed rapidly, with no residual contractile activity until the next on-demand administration. However, a significant limitation of peptide NK2 agonists is their poor selectivity for NK2 over NK1 receptors. Despite its weak ability to displace radiolabeled substance P from recombinant NK1 receptors, NKA is a potent NK1 receptor agonist in functional assays and binds with subnanomolar affinity to a septide-sensitive site on NK1 receptors [13–15]. Activation of NK1 receptors by NKA and related analogs can cause unwanted effects including hypotension [9, 12, 16] and emesis [17], and so it is important to identify NK2 agonists with a high degree of selectivity for NK2 receptors over septide-sensitive sites on NK1 receptors.

The present studies characterized a series of peptide agonists to explore how various structural changes influence NK2 receptor affinity, potency, efficacy and selectivity. It is known that the truncated peptide, NKA(4–10), has greater potency for NK2 receptors, and that substitution of Met^10^ with Nle yields a more selective but less potent agonist than NKA [18]. Other substitutions that improved potency and selectivity were replacing Ser^5^ with Arg [19], or Lys [20], replacing Gly^8^ with β-Ala [21], and Leu^9^ with MeLeu [22]. Multiple substitutions, such as [MeLeu^9^,Nle^10^]NKA(4–10), [Lys^5^,MeLeu^9^,Nle^10^]NKA(4–10) and [Lys^5^,Tyr^7^,MeLeu^9^,Nle^10^]NKA(4-10), also conferred greater potency, but not affinity [20, 22]. A limitation of these studies is that they employed native tissues that are not pure monoreceptor systems, and so interpretation is complicated by the presence of other receptors (including NK1) that may contribute to NK2 receptor-mediated responses [18, 20]. They also did not examine the effects of these modifications on affinity and agonist efficacy at the septide site on NK1 receptors. The present studies extend these observations by using recombinant cell lines expressing either human NK2 or NK1 receptors for radiolabeled binding and functional studies. Substitutions known to improve potency were used as a basis to examine additional modifications, generating novel peptides that were not previously studied. The effects of these amino acid modifications on the potency and affinity of the peptides for the septide site of the NK1 receptor were also examined for the first time.

## Materials and methods

### Materials

NKA and the peptide analogs of NKA listed in Table 1 were synthesized by Genscript (Piscataway, USA) to a purity ≤95%. NKA and substance P were purchased from Sigma Aldrich, (St. Louis, USA); septide was purchased from Bachem (Bubendorf, Switzerland, EU). [^3^H]-Septide was custom synthesized from Quotient Bioresearch (Cardiff, UK). [^125^I]-NKA, and Microscint 20, were obtained from Perkin Elmer (Boston, USA). F12K medium, Geneticin (G418) and Lipofectamine 2000 were obtained from LifeTechnologies (Carlsbad, USA). Phosphate buffered saline (PBS) was from Lonza (Walkersville, MD, USA) and EDTA from Life Technologies (New York, USA). FuGENE HD Transfection Reagent was obtained from Promega (Madison, USA). The following reagents were obtained from Sigma Aldrich (St. Louis, USA): DMSO, Pluronic F-127, HEPES, bacitracin, SIGMA*FAST* Protease Inhibitor Tablets, bovine serum albumin (BSA), polyethileneimine (PEI), NaCl, 3-isobutyl-1-methylxantine (IBMX), and probenecid. The CRE/CREB Reporter Assay Kit, Firefly Luciferase reagent, and Renilla Luciferase reagent were purchased from BPS Bioscience (San Diego, USA).

**Table 1:** Test compounds.

Common Name	Amino Acid Sequence
NKA	His-Lys-Thr-Asp-Ser-Phe-Val-Gly-Leu-Met-NH_2_
NKA(4–10)	Asp-Ser-Phe-Val-Gly-Leu-Met-NH_2_
Substance P	Arg-Pro-Lys-Pro-Gln-Gln-Phe-Phe-Gly-Leu-Met-NH_2_
[Lys^5^,MeLeu^9^, Nle^10^]-NKA(4–10)	Asp-Lys-Phe-Val-Gly-Leu(NMe)-Nle-NH_2_
[Lys^5^]-NKA(4–10)	Asp-Lys-Phe-Val-Gly-Leu-Met-NH_2_
[Lys^5^,β-Ala^8^]-NKA(4–10)	Asp-Lys-Phe-Val-[β-Ala]-Leu-Met-NH_2_
[Lys^5^,MeLeu^9^]-NKA(4–10)	Asp-Lys-Phe-Val-Gly-(NMe-Leu)-Met-NH_2_
[Lys^5^,Nle^10^]-NKA(4–10)	Asp-Lys-Phe-Val-Gly-Leu-Nle-NH_2_
[Lys^5^,β-Ala^8^,Nle^10^]-NKA(4–10)	Asp-Lys-Phe-Val-[β-Ala]-Leu-Nle-NH_2_
[Arg^5^,MeLeu^9^,Nle_10_]-NKA(4–10)	Asp-Arg-Phe-Val-Gly-(NMe-Leu)-Nle-NH_2_
[Arg^5^]-NKA(4–10)	Asp-Arg-Phe-Val-Gly-Leu-Met-NH_2_
[Arg^5^,β-Ala^8^]-NKA(4–10)	Asp-Arg-Phe-Val-[β-Ala]-Leu-Met-NH_2_
[Arg^5^,MeLeu^9^]-NKA(4–10)	Asp-Arg-Phe-Val-Gly-(NMe-Leu)-Met-NH_2_
[Arg^5^,Nle^10^]-NKA(4–10)	Asp-Arg-Phe-Val-Gly-Leu-Nle-NH_2_
[Arg^5^,β-Ala^8^,Nle^10^]-NKA(4–10)	Asp-Arg-Phe-Val-[β-Ala]-Leu-Nle-NH_2_
[β-Ala^8^]-NKA(4–10)	Asp-Ser-Phe-Val-[β-Ala]-Leu-Met-NH_2_

### Cell cultures

Human recombinant NK2 and NK1 receptors were generated using stably transfected CHO cells (CHO-hNK2 and CHO-hNK1, respectively). Human NK2 or NK1 receptor expressing vectors from Genecopeia (Rockville, USA) were transfected in CHO cells using the standard FuGENE protocol and cells were selected using 450 μg/mL geneticin (G418). Clones expressing the receptors were selected by functional coupling to calcium using FLIPR and a single clone for each receptor subtype was selected for expansion and stable cell line generation. Cells were cultured in a humidified incubator with 5% CO_2_ in F12K medium containing 10% heat inactivated FBS and 450 μg/mL geneticin and passaged on reaching 80–90% confluence.

### Radioligand binding

#### Membrane preparation

Protein expression was induced by addition of 5 mM sodium butyrate to the culture medium. After 16 h, the medium was removed and the cells were washed with PBS (calcium and magnesium-free) and detached. The cell suspension was collected, placed on ice and centrifuged for 5 min at 4°C and 1200 rpm in a Beckman GS6R centrifuge. After removal of the supernatant the cell pellet was washed and collected by re-suspension in PBS and centrifugation. The final pellet was weighed and frozen at -80°C until use.

Frozen pellets were thawed and homogenized in 10 volumes (w/v) of membrane preparation buffer (50 mM HEPES pH 7.4, 1 mM EDTA, 50 μg/mL bacitracin and protease inhibitors) using a Polytron Ultraturrax (twice for 15 s per cycle). The homogenate was centrifuged for 20 min at 4°C and 18500 rpm in a SL-50T Sorvall rotor and the pellet was re-suspended in membrane preparation buffer and re-homogenized as before. After centrifugation for 20 min at 4°C and 18500 rpm, the pellets were re-suspended in 5 volumes of membrane preparation buffer and divided into aliquots before freezing at -80°C. Protein concentration was determined using BioRad Protein Assay (Milan, Italy) with a BSA standard curve.

#### Filtration assays

Stock solutions of test compounds (10 mM) were prepared in DMSO and stored at -20°C until use. Further dilutions were performed in DMSO to provide an 11-point concentration response curve spanning final concentrations from 0.01 nM to 10 μM. Radioligand binding experiments were performed immediately after transferring 2 μL of each concentration of test compound to a 96-well plate. Each well contained a final volume of 200 μL buffer (50 mM HEPES, 3 mM MnCl_2_, 0.02% BSA, 0.02% Pluronic F-127 and 50 μg/mL bacitracin, pH 7.4). All reactions (except for [^3^H]-Septide saturation curve) were stopped by rapid filtration through Unifilter-96 GF/C filter plates pre-soaked for one hour in 0.5% PEI followed by 3 washings with 1 mL ice-cold 0.9% NaCl using a Packard cell harvester. After drying for 1 h at 40°C, 50 μL of Microscint-20 was added to each filter plate and bound radioactivity was measured using a Microplate TopCount (Packard C9912). [^3^H]-Septide saturation reactions were terminated by rapid filtration through GF/B filter paper pre-soaked in PEI 0.5% (w/v) solution and washed with 1 mL of ice cold 0.9% NaCl before filtration on a Brandel Harvester. Filters were washed 4 times with 1 mL ice cold 0.9% NaCl and placed into pico vials with 4 mL of Filter Count. The radioligand concentration was determined by measurement of 50 μL of [^125^I]-NKA, or 100 μL of [^3^H]-Septide, mixed with 3 mL of Filter Count using a β-Counter TriCarb 2900.

#### [^125^I]-NKA binding to human recombinant NK2 receptors

100 μL of [^125^I]-NKA (specific activity 81.4 TBq/mmol) was incubated with 100 μL of the CHO-hNK2 membrane suspension under the following conditions: to determine protein linearity, 0.1 nM [^125^I]-NKA was incubated with increasing concentrations of CHO-hNK2 membranes (1, 3, 10 and 30 μg/well) at 23°C for 2 h; to examine association kinetics, 0.1 nM [^125^I]-NKA was incubated with CHO-hNK2 membranes (6 μg/well) at 23°C for a range of durations from 10 to 240 min; in the saturation study, final concentrations of [^125^I]-NKA and NKA from 0.02 to 5 nM (1 part hot/4 parts cold) were incubated with CHO-hNK2 membranes (6 μg/well) at 23°C for 3 h; in competition binding experiments, test compounds were incubated with 0.1 nM [^125^I]-NKA and CHO-hNK2 membranes (6 μg/well) at 23°C for 3 h. Total binding was defined by the addition of 2 μL DMSO, and nonspecific binding was defined by the addition of 2 μL of 100 μM NKA (1 μM final concentration).

#### [^3^H]-Septide binding to human recombinant NK1 receptors

100 μL of [^3^H]-Septide (specific activity 3.9 TBq/mmol) was incubated with 100 μL of CHO-hNK1 membrane suspension under the following conditions: to determine protein linearity, 4 nM [^3^H]-Septide was incubated with increasing concentrations of CHO-hNK1 membranes (10, 15, 20, 25 and 30 μg/well) at 23°C for 90 min; to examine association kinetics, 5 nM [^3^H]-Septide was incubated with CHO-hNK1 membranes (17 μg/well) at 23°C for a range of durations from 2 to 120 min; in the saturation study, final concentrations of [^3^H]-Septide from 0.1 to 100 nM were incubated with CHO-hNK1 membranes (20 μg/well) at 23°C for 2 h; in competition binding experiments, test compounds were incubated with 5.0 nM [^3^H]-Septide and CHO-hNK1 membranes (20 μg/well) at 23°C for 1 h. In the saturation binding studies using different concentrations of [^3^H]-Septide, incubation for 2 h were carried out to allow all concentrations, including the lowest, to reach equilibrium. The association study with 5 nM [^3^H]-Septide confirmed that equilibrium was reached after 1 h and, therefore, 1 h was selected for the displacement binding studies. Total binding was defined by the addition of 2 μL of DMSO, and nonspecific binding was defined by the addition of 2 μL of 100 μM septide (1 μM final concentration).

### Functional assays

#### Intracellular calcium response

The agonist efficacy of the test compounds at recombinant human NK1 or NK2 receptors expressed in CHO cells was assessed by measuring the intracellular calcium response using the calcium-sensitive dye Fluo-4 AM (Eugene, OR, USA) and a Fluorometric Imaging Plate Reader (FLIPR, Molecular Devices, CA, USA). CHO-hNK1 and CHO-hNK2 cells were seeded into black walled clear-bottom 384-well plates at a density of 10,000 and 15,000 cells per well in 50 μL culture media, respectively, and grown overnight at 37°C in a humidified CO_2_-incubator. Cells were washed in washing buffer using an EMBLA 384 plate washer, leaving 20 μL of buffer per well after the final aspiration. Cells were then incubated at 37°C with the cytoplasmic Ca^2+^ indicator Fluo-4 AM (final concentration 1 μM) in assay buffer (20 mM HEPES, 145 mM NaCl, 5 mM KCl, 5.5 mM glucose, 1 mM MgCl_2_ and 2 mM CaCl_2_, pH 7.4, 0.05% Pluronic F-127 and 0.1% BSA) containing 2.5 mM probenecid for 45–60 min (cell loading). Cells were then washed 3 times in washing buffer (20 mM HEPES, 145 mM NaCl, 5 mM KCl, 5.5 mM glucose, 1 mM MgCl_2_ and 2 mM CaCl_2_, pH 7.4 and 2.5 mM probenecid) using an EMBLA 384 plate washer, leaving 30 μL of buffer in each well after the last wash. Loaded cell plates were transferred to the FLIPR and calcium responses were monitored as described below. For quality control, in each compound plate included the reference standards NKA and substance P. Eleven concentrations of the test compounds were evaluated for their ability to increase intracellular calcium levels with respect to the agonist reference standard (NKA or substance P), and the EC_50_ value was calculated. The range of final concentrations tested was 0.169 nM to 10 μM, or 1.69 pM to 100 nM, depending on compound potency. Concentration response curves of test compounds were generated in duplicate on at least two different occasions from the same stock solutions. Test solutions were prepared from 10 mM stock solutions in DMSO and 1 μL of each solution was stamped into V-bottom assay plates containing 49 μL assay buffer. The final concentration of DMSO was 0.5% in each well.

Responses were measured as peak fluorescence intensity (FI) minus basal FI and expressed as a percentage of the maximal response induced by NKA for the NK2 receptor, or substance P for the NK1 receptor. Maximal responses to NKA and substance P were calculated by GraphPad Prism v5 concentration response curve fitting (see Data Analysis).

#### Cyclic AMP stimulation

Four peptides that spanned the range of NK2 receptor agonist potency on the intracellular calcium response (NKA, substance P, [Lys^5^,MeLeu^9^, Nle^10^]-NKA(4–10) and β-Ala^8^]-NKA(4–10)) were selected for examination in assays of Gs coupling to NK2 and NK1 receptors to stimulate cyclic AMP (cAMP) production. CHO-hNK2 and CHO-hNK1 cells were transiently transfected with a CRE-LUC reporter construct using Lipofectamine 2000. At 24 h post-transfection, cells were plated at a density of 20,000 cells/well in a 96-well culture plate. After 24 h, cells were stimulated with culture medium containing 0.5 mM IBMX and 7 concentrations of the test compounds in the range 0.1 nM—1 μM or 0.19 pM—30 nM, depending on the potency of the compound. Luciferase expression was measured after 5 h of incubation at 37°C (in the presence of 5% CO_2_) by adding Firefly Luciferase reagent, followed by Renilla Luciferase reagent. Luminescence was measured by using an Envision Multilabel flash lamp reader after addition of the first and second reagents. Data were normalized by calculating the ratio of the Firefly luminescence from the CRE reporter to the Renilla luminescence from the control Renilla Luciferase vector.

### Data analysis

#### Radioligand binding

Raw data were expressed as total binding (TB), or nonspecific binding (NSB) measured in the presence of excess NKA, septide or substance P. Specific binding (SB) was derived by subtraction of NSB from TB. Saturation experiment curve fitting and K_D_ estimates were performed using Global Fitting Analysis in GraphPad Prism v5 (GraphPad Software Inc, California, USA) by comparing the results of One-Site and Two-Site models to simultaneously fit both TB and NSB (the X axis was radioligand concentration in nM and the Y axis was TB and NSB expressed in dpm or pmol/mg protein). The goodness of fit between One-Site and Two-Site models was examined using Akaike’s Information Corrected Criteria (AICC; [23]) in SAS v9.4 (SAS Institute, Cary NC) in order to select the best model for the data generated. K_D_ and B_max_ were determined by analysis of the saturation data using the Total and Nonspecific Binding equation in GraphPad Prism v5. Data from displacement experiments were plotted as the log [inhibitor concentration] (X axis) versus response expressed as TB in cpm (Y axis). Curve fitting, IC_50_ values and Hill slope were determined using a four-parameter logistic model in GraphPad Prism v5. K_i_ value estimates were made using the One Site-fit K_i_ equation in GraphPad Prism v5, by applying the Cheng-Prusoff equation [24]: K_i_ = IC_50_ /1+([L]/KD) where [L] is the radioligand concentration in the displacement assay, and K_D_ is the dissociation constant of the radioligand determined in the saturation experiment.

Mean data ± standard deviations (SD) for pIC_50_ (-log IC_50_), slope and pK_i_ (-log of K_i_) were calculated from at least two independent experiments with each concentration tested in duplicate. The displacement curves were superimposable with standard deviations less than 0.3.

#### Functional assays

Agonist concentration response curves were analyzed by GraphPad Prism v5 using a four-parameter logistic equation:

R=Max+Min−Max1+(xEC50)^n

where R is the agonist response, X the agonist concentration, Max and Min are the maximal and the basal effect, EC_50_ is the concentration producing 50% of the maximal effect and n is the Hill slope. Results are reported as mean ± SD of the calculated potency value pEC_50_ (-log EC_50_) and % of the maximal effect of the reference standard agonist from at least two independent experiments with each concentration tested in duplicate.

## Results

### Affinity, potency and efficacy of NK2 receptor agonists for human recombinant NK2 receptors

#### Characterization of [^125^I]-NKA binding to human NK2 receptors expressed in CHO cell membranes

**Fig 1:**
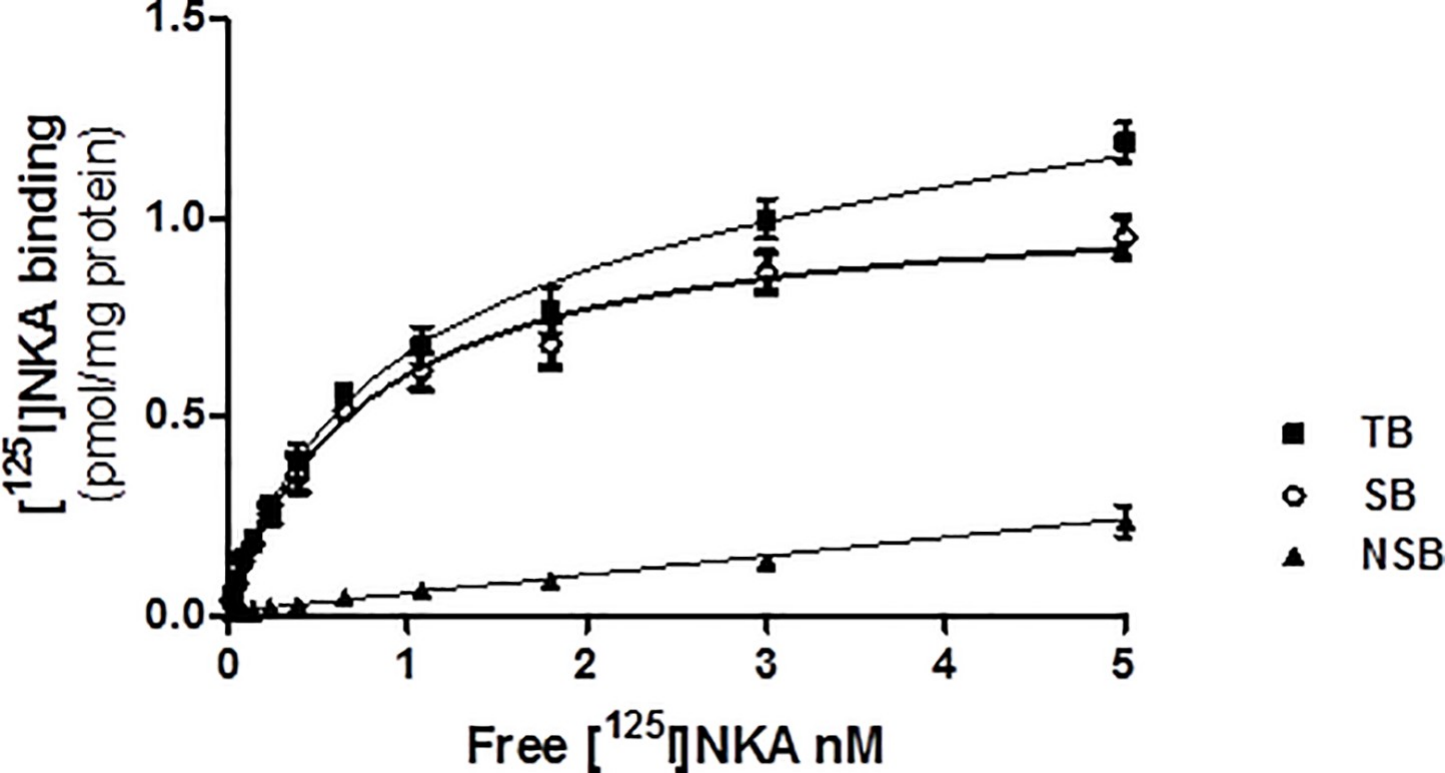
Saturation binding of [^125^I]-NKA to human recombinant NK2 receptors. [^125^I]-NKA binding to human NK2 receptors at increasing radioligand concentrations. Saturation data were subjected to nonlinear regression analysis. Values are means ± SD of triplicate determinations of TB and duplicates of NSB at each radioligand concentration. TB: total binding; SB: specific binding; NSB: nonspecific binding.

Specific [^125^I]-NKA binding increased linearly with protein content up to 10 μg per well. Radioligand depletion was <10% and specific binding was 89–95% of total binding between 3 and 10 μg protein per well. A protein concentration of 6 μg per well was used for the experiments. Since association of 0.1 nM [^125^I]-NKA reached equilibrium after 3 h at 23°C, an incubation time of 3 h was selected. AICC analysis showed that the One-Site model provided a better goodness of fit for the individual data than the Two-Site model (AICC = -83.9 for the One-Site model vs -81.5 for the Two-Site model), suggesting that saturation data were consistent with a single population of binding sites (see Fig 1). [^125^I]-NKA bound to recombinant human NK2 receptors with a K_D_ of 0.74 nM (pK_D_ = 9.13) and B_max_ of 1.0 pmol mg^-1^. Displacement experiments used 0.1 nM of [^125^I]-NKA.

#### Displacement of [^125^I]-NKA binding

**Fig 2:**
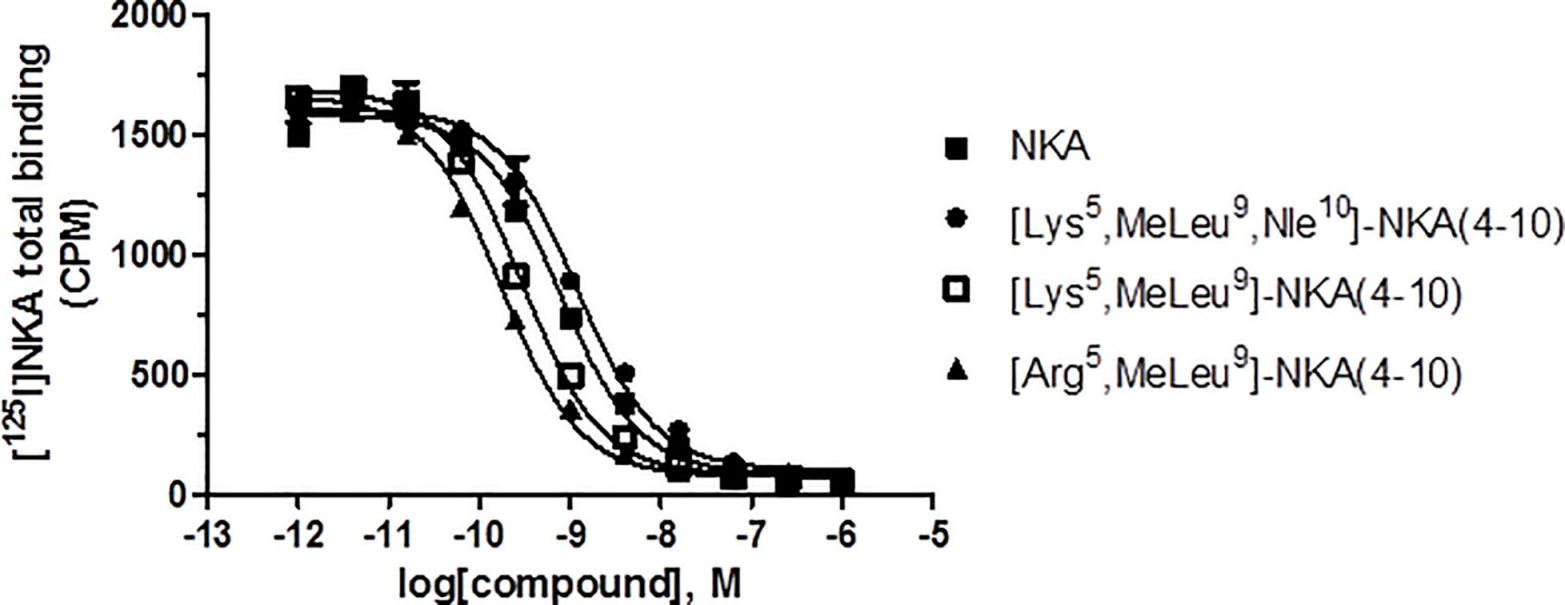
Representative curves showing displacement of [^125^I]-NKA binding to human recombinant NK2 receptors by NKA, [Lys^5^,MeLeu^9^,Nle^10^]-NKA(4–10), [Lys^5^,MeLeu^9^]-NKA(4–10) and [Arg^5^,MeLeu^9^]-NKA(4–10). Displacement curves were fitted using a one-site model. Values are means ± SD from a representative experiment performed in duplicate.

All test compounds competed for [^125^I]-NKA binding to human NK2 receptors, with [Arg^5^,MeLeu^9^]-NKA(4-10) and [Lys^5^,MeLeu^9^]-NKA(4–10) showing the highest affinity and [Lys^5^,β-Ala^8^,Nle^10^]-NKA(4–10) the lowest (pK_i_ range from 9.90 to 7.79). Most Hill slopes approached unity, ranging from approximately -0.8 to -0.9. However, for [β-Ala^8^]-NKA(4–10) and [Arg^5^,β-Ala^8^]-NKA(4–10) the calculated Hill slopes were significant lower than unity (p<0.05); therefore, for these two compounds only, the pIC_50_ value is reported. Displacement curves for NKA, [Lys^5^,MeLeu^9^,Nle^10^]-NKA(4–10), [Lys^5^,MeLeu^9^]-NKA(4-10) and [Arg^5^,MeLeu^9^]-NKA(4–10) are shown in Fig 2 and binding affinities of all compounds tested are shown Table 2.

**Table 2:** Displacement of [^125^I]-NKA binding, intracellular calcium response and stimulation of cyclic AMP in CHO cells expressing human recombinant NK2 receptors.

Compound	[^125^I]-NKA binding		Intracellular Ca^2+^		cAMP stimulation	
	Slope	pK_i_	pEC_50_	% Max	pEC_50_	% Max
NKA	-0.84 ± 0.04	9.29 ± 0.19	9.32 ± 0.22	102.3 ± 4.5	10.49 ± 0.36	106.2 ± 18.3
Substance P	ND	7.15 ± 0.37	98.3 ± 4.4	8.43 ± 0.23	87.7 ± 3.87
[Lys^5^,MeLeu^9^,Nle^10^]-NKA(4-10)	-0.86 ± 0.07	9.06 ± 0.12	9.83 ± 0.38	101.4 ± 3.0	10.62 ± 0.53	107.4 ± 7.36
[β-Ala^8^]-NKA(4–10)	-0.72 ± 0.11	8.00* ± 0.17	9.06 ± 0.29	102.2 ± 7.4	10.63 ± 0.29	84.6 ± 13.1
NKA(4–10)	-0.87 ± 0.03	8.63 ± 0.14	8.99 ± 0.10	107.4 ± 6.9	ND	
[Arg^5^,MeLeu^9^]-NKA(4-10)	-0.90 ± 0.07	9.90 ± 0.12	9.79 ± 0.17	111.5 ± 3.7		
[Lys^5^,MeLeu^9^]-NKA(4–10)	-0.84 ± 0.06	9.70 ± 0.16	9.84 ± 0.55	88.2 ± 11.4		
[Arg^5^]-NKA(4–10)	-0.81 ± 0.11	9.51 ± 0.01	9.72 ± 0.13	106.7 ± 2.6		
[Lys^5^]-NKA(4–10)	-0.87 ± 0.05	9.42 ± 0.18	10.05 ± 0.32	95.6 ± 0.1		
[Arg^5^,MeLeu^9^,Nle^10^]-NKA(4-10)	-0.86 ± 0.09	9.23 ± 0.19	10.08 ± 0.04	89.0 ± 15.5		
[Arg^5^,β-Ala^8^]-NKA(4–10)	-0.70 ± 0.05	9.00* ± 0.22	9.82 ± 0.14	106.9 ± 8.9		
[Arg^5^,Nle^10^]-NKA(4–10)	-0.83 ± 0.04	8.76 ± 0.15	9.70 ± 0.11	98.9 ± 3.9		
[Lys^5^,β-Ala^8^]-NKA(4–10)	-0.78 ± 0.00	8.68 ± 0.10	9.76 ± 0.32	92.0 ± 3.8		
[Lys^5^,Nle^10^]-NKA(4–10)	-0.74 ± 0.09	8.56 ± 0.09	9.42 ± 0.28	90.0 ± 0.0		
[Arg^5^,β-Ala^8^,Nle^10^]-NKA(4–10)	-0.75 ± 0.07	8.16 ± 0.13	8.94 ± 0.32	81.4 ± 1.2		
[Lys^5^,β-Ala^8^,Nle^10^]-NKA(4–10)	-0.79 ± 0.07	7.79 ± 0.06	8.52 ± 0.41	83.1 ± 25.5		

Data are means ± SD from 2 experiments performed in duplicate (radioligand binding), 2–6 experiments performed in duplicate (intracellular Ca^2+^), or 3–4 experiments performed in duplicate (cAMP stimulation). For Ca^2+^ and cAMP assays, % Max refers to the maximal response observed with NKA. ND: not determined.

*Binding data for [β-Ala^8^]-NKA(4–10) and [Arg^5^,β-Ala^8^]-NKA(4–10) are expressed as pIC_50_.

#### Intracellular calcium response in CHO cells expressing human recombinant NK2 receptors

**Fig 3:**
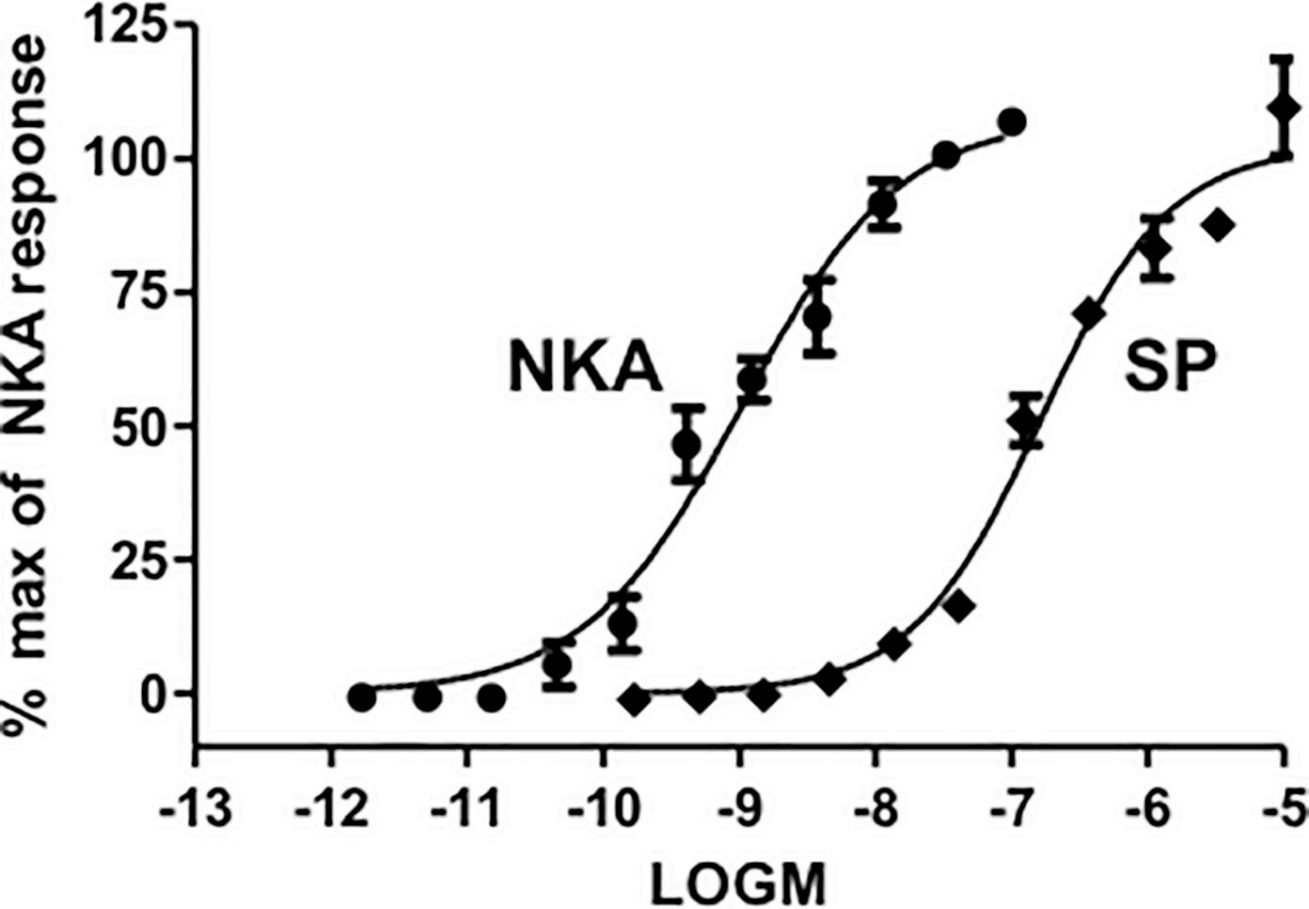
Concentration-response curves for NKA and substance P on intracellular calcium response using human cloned NK2 receptors. Data are expressed as % of maximal response to NKA (30 nM). Each data point is the mean ± SD of data from an individual experiment performed in duplicate.

**Fig 4:**
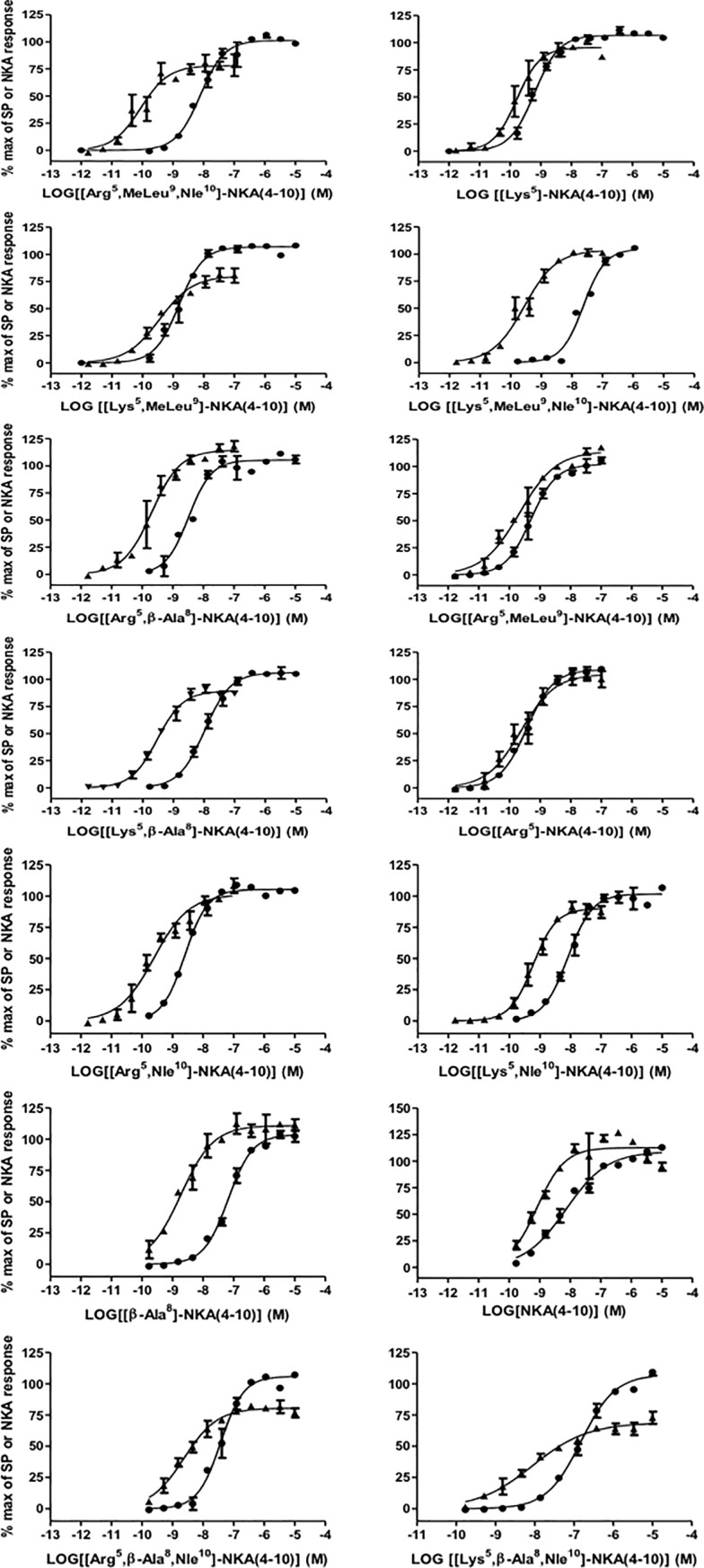
Concentration-response curves for individual test compounds on intracellular calcium levels using human cloned NK2 and NK1 receptors. Data are expressed as % of maximal response to 30 nM NKA (for NK2 receptors) or 30 nM substance P (for NK1 receptors). Triangles are NK2 receptors; circles are NK1 receptors. Each data point is the mean ± SD of data from an individual experiment performed in duplicate.

**Fig 5:**
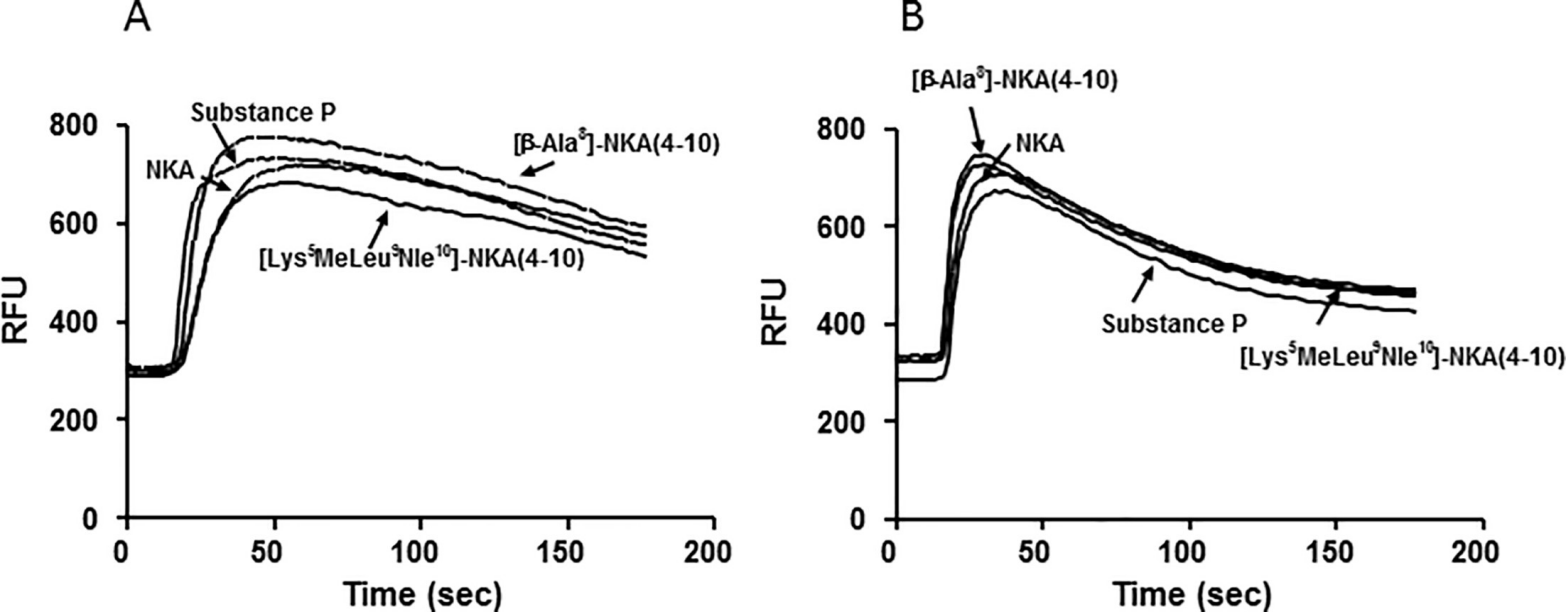
Representative calcium traces for NKA, substance P, [Lys^5^,MeLeu^9^,Nle^10^]-NKA(4–10) and [β-Ala^8^]-NKA(4–10) using human recombinant NK2 and NK1 receptors. Compounds were tested at the EC_50_ concentration. Data are expressed as relative fluorescence units (RFU) measured over a period of 200 seconds. A: NK2 receptors, B: NK1 receptors.

Both NKA and substance P were full agonists at NK2 receptors as determined by the intracellular calcium response; NKA was approximately 150-fold more potent than substance P (Fig 3; Table 2). All test compounds elicited concentration-dependent calcium responses in CHO cells expressing NK2 receptors (Fig 4). With the possible exception of [Arg^5^,β-Ala^8^,Nle^10^]-NKA(4–10), all compounds were full agonists under the assay conditions employed. [Arg^5^,MeLeu^9^,Nle^10^]-NKA(4-10) was the most potent agonist and substance P the least potent (pEC_50_ range 10.08 to 7.15). Representative calcium traces are shown for NKA, substance P, [Lys^5^,MeLeu^9^,Nle^10^]-NKA(4–10) and [β-Ala^8^]-NKA(4–10) at the EC_50_ concentration in Fig 5A. All compounds tested displayed a similar calcium profile over time at NK2 receptors.

**Fig 6:**
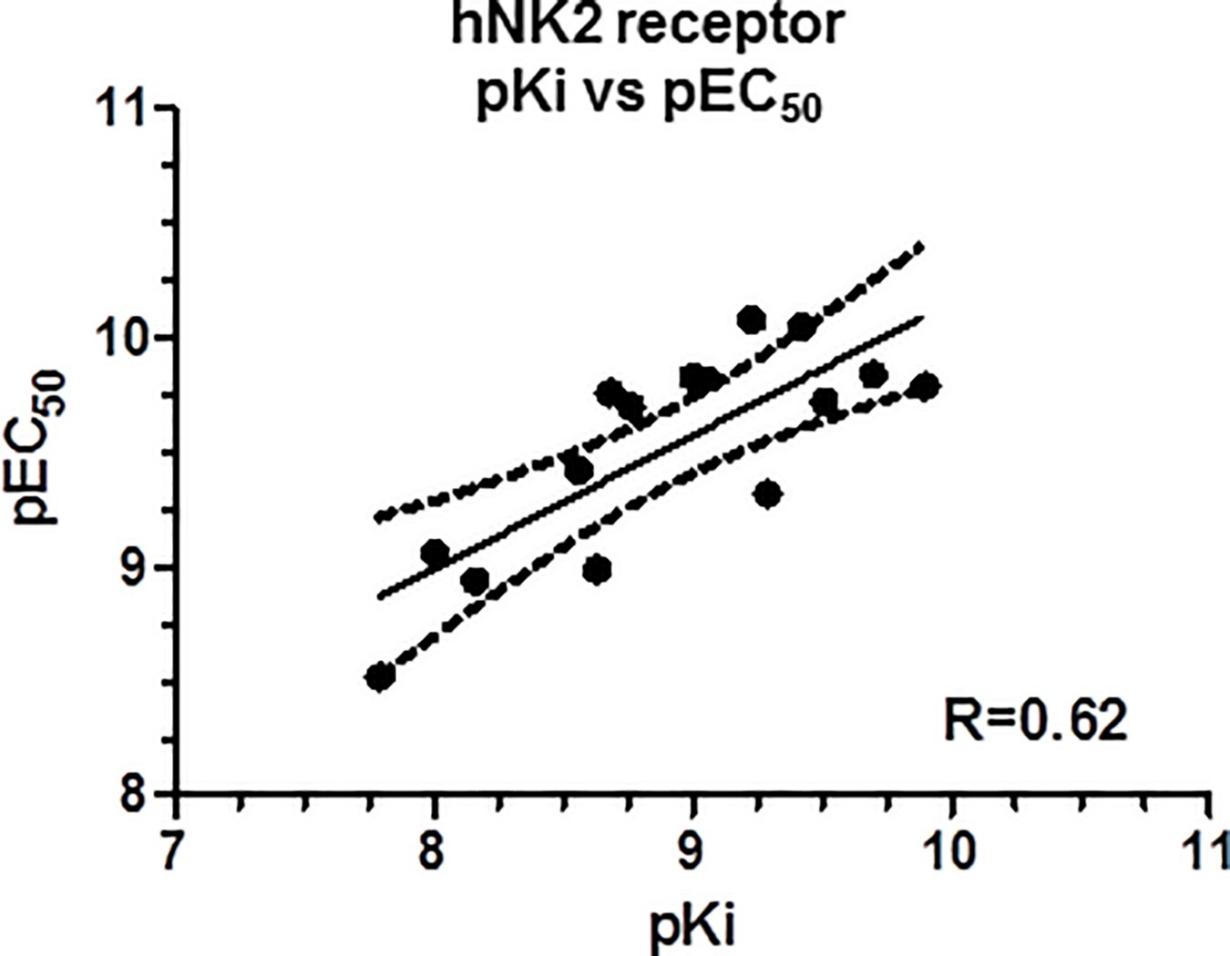
Correlation between displacement of [^125^I]-NKA binding (pKi) and intracellular calcium response (pEC_50_) at human recombinant NK2 receptors expressed in CHO cells. Scatterplot and regression analysis of pKi versus pEC_50_ values at human recombinant NK2 receptors for a series of NK2 agonists. Solid line: regression analysis; dotted lines: 95% confidence intervals. For [Arg^5^,β-Ala^8^]-NKA(4–10) and [β-Ala^8^]-NKA(4–10), pIC_50_ values were used.

The pKi values for displacement of [^125^I]-NKA binding and pEC_50_s for the intracellular calcium response in CHO cells expressing human recombinant NK2 receptors were normally distributed (Shapiro-Wilk test, p>0.05). There was a linear correlation between pKi and pEC_50_ values (R^2^ = 0.62; Fig 6).

### Cyclic AMP Stimulation in CHO cells expressing human recombinant NK2 receptors

**Fig 7:**
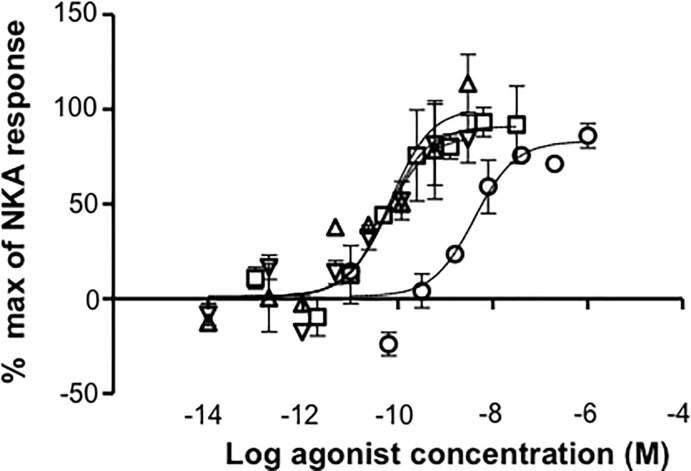
Concentration-response curves for stimulation of cyclic AMP by selected compounds using human cloned NK2 receptors. Data are expressed as % of maximal response to 30 nM NKA. Triangles are [Lys^5^,MeLeu^9^,Nle^10^]-NKA(4–10); inverted triangles are [β-Ala^8^]-NKA(4–10); squares are NKA; circles are substance P. Data are the mean ± SD of one representative experiment performed in duplicate.

[Lys^5^,MeLeu^9^,Nle^10^]-NKA(4–10), [β-Ala^8^]-NKA(4–10) and NKA potently stimulated cAMP production via Gs coupling to NK2 receptors; substance P was the weakest agonist tested (Fig 7; Table 2). All four peptides were full agonists.

### Affinity, potency and efficacy of NK2 receptor agonists at human recombinant NK1 receptors

#### Characterization of [^3^H]-Septide binding to human NK1 receptors expressed in CHO cell membranes

**Fig 8:**
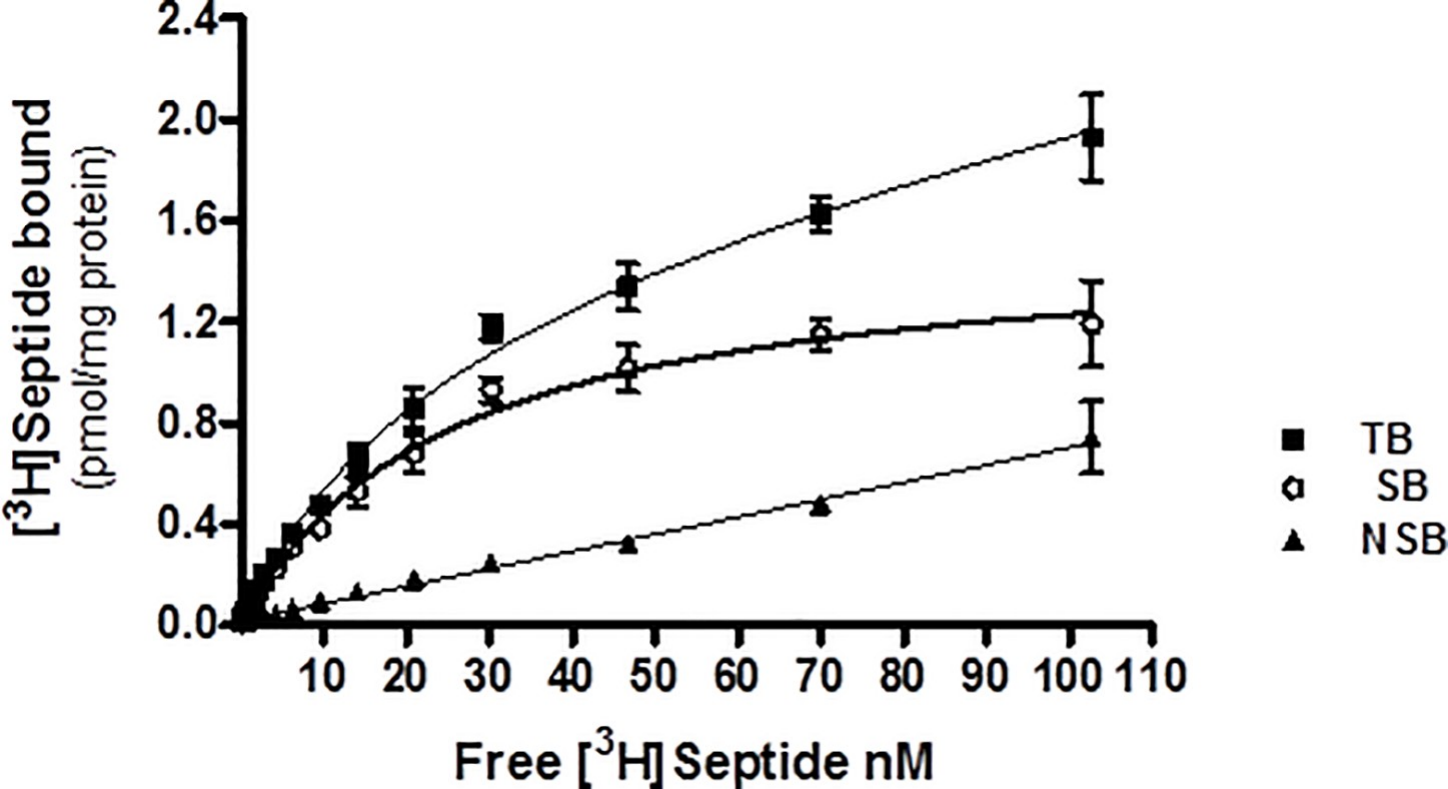
Saturation binding of [^3^H]-Septide to human recombinant NK1 receptors. [^3^H]-Septide binding to human NK1 receptors expressed in CHO membranes with increasing radioligand concentrations. Saturation data were subjected to nonlinear regression analysis. Values are means ± SD of triplicate determinations of TB and duplicates of NSB at each radioligand concentration. TB: total binding; SB: specific binding; NSB: nonspecific binding.

Specific [^3^H]-Septide binding increased linearly with protein content up to 30 μg per well. A protein concentration of 20 μg per well was used for displacement experiments. Specific binding was 83–86% of total binding. Association of 5 nM [^3^H]-Septide reached equilibrium after 30 min and remained stable after 120 min at 23°C. Incubation times of 120 and 60 min were selected for the saturation and competition binding experiments, respectively. AICC analysis showed a better goodness of fit for the individual data fitted using a One-Site model than a Two-Site model (AICC = -145.1 for One-Site vs -140.3 for Two-Site model), suggesting that the saturation data were consistent with a single population of binding sites (Fig 8). [^3^H]-Septide bound to the human cloned NK1 receptor with a K_D_ of 22.5 nM (pK_D_ = 7.65) and B_max_ of 1.3 pmol mg^-1^ protein. Displacement experiments used 5 nM of [^3^H]-Septide.

### Displacement of [^3^H]-Septide binding

**Fig 9:**
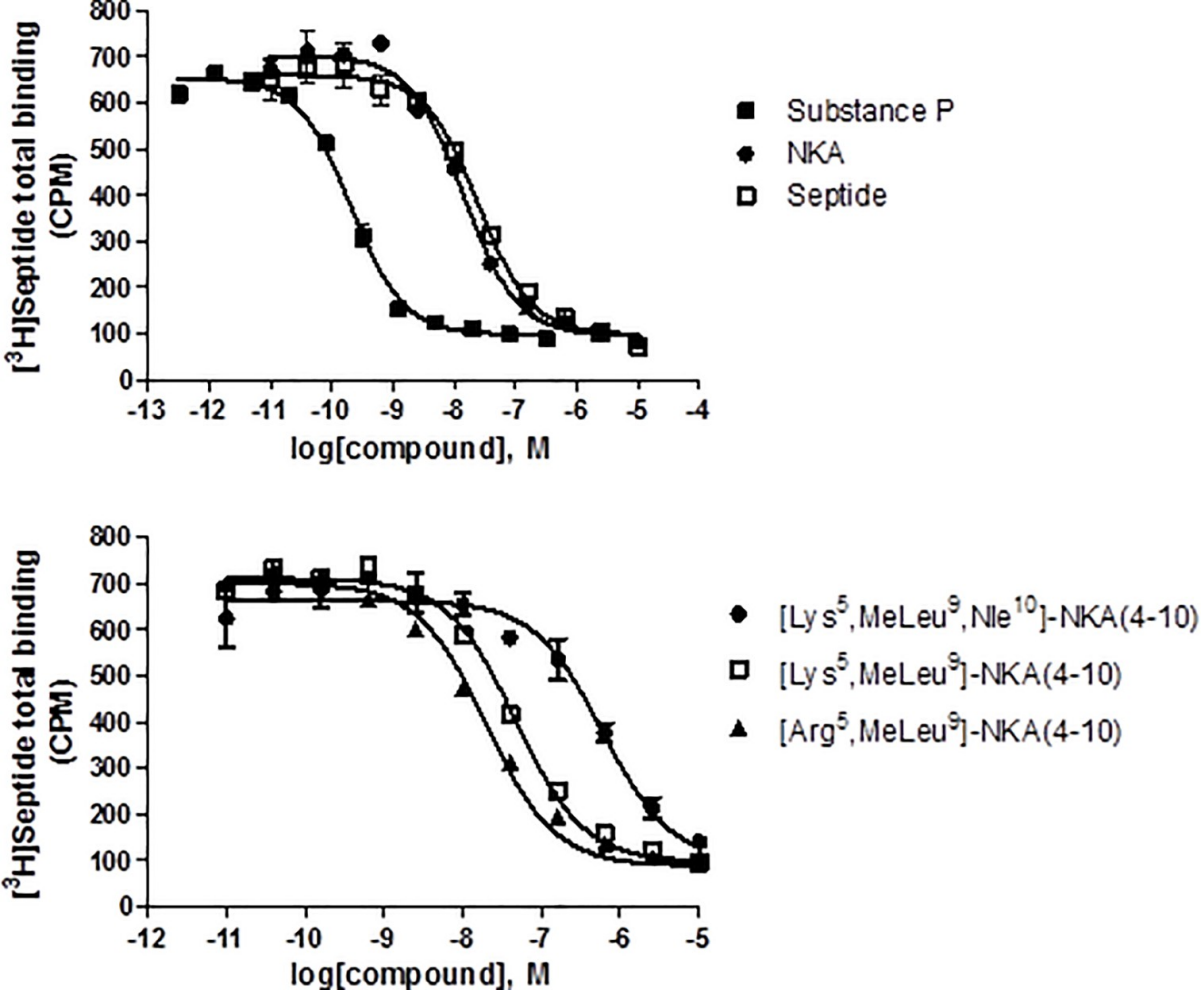
Representative curves showing displacement of [^3^H]-septide binding to human recombinant NK1 receptors by substance P, NKA, septide, [Lys^5^,MeLeu^9^,Nle^10^]-NKA(4–10), [Lys^5^,MeLeu^9^]-NKA(4–10) and [Arg^5^,MeLeu^9^]-NKA(4–10). Displacement curves were fitted using a one-site model. Values are means ± SD from a representative experiment performed in duplicate.

All compounds competed for [^3^H]-Septide binding to human NK1 receptors. Substance P had the highest affinity and [Lys^5^,β-Ala^8^,Nle^10^]-NKA(4–10) the lowest affinity (pKi range 9.83 to 5.51). All compounds exhibited Hill slopes close to unity (between approximately -0.8 and -1.1). Displacement curves for substance P, NKA, septide, [Lys^5^,MeLeu^9^,Nle^10^]-NKA(4–10), [Lys^5^,MeLeu^9^]-NKA(4-10) and [Arg^5^,MeLeu^9^]-NKA(4–10) are shown in Fig 9. Binding data for all compounds tested at the septide site of the human NK1 receptor are shown in Table 3.

**Table 3:** Displacement of [^3^H]-Septide binding, intracellular calcium response and stimulation of cyclic AMP in CHO cells expressing human recombinant NK1 receptors.

Compound	[^3^H]-Septide binding		Intracellular Ca^2+^		cAMP stimulation	
	Slope	pK_i_	pEC_50_	% Max	pEC_50_	% Max
NKA	-0.95 ± 0.02	8.00 ± 0.10	9.28 ± 0.39	108.3 ± 5.9	10.04 ± 0.42	100.5 ± 13.6
Substance P	-1.13 ± 0.05	9.83 ± 0.08	10.48 ± 0.38	101.9 ± 3.3	9.75 ± 0.29	113.3 ± 13.0
[Lys^5^,MeLeu^9^,Nle^10^]-NKA(4-10)	-0.95 ± 0.15	6.23± 0.13	7.81 ± 0.46	107.6 ± 3.1	8.75 ± 0.25	94.3 ± 21.6
[β-Ala^8^]-NKA(4–10)	-0.95 ± 0.12	5.68 ± 0.11	7.33 ± 0.43	106.0 ± 11.5	8.25 ± 0.71	97.3 ± 24.8
NKA(4–10)	-0.92 ± 0.13	6.83 ± 0.16	8.40 ± 0.39	117.4 ± 12.8	ND	
[Arg^5^,MeLeu^9^]-NKA(4-10)	-0.75 ± 0.03	7.68 ± 0.16	9.52 ± 0.29	99.9 ± 13.3		
[Lys^5^,MeLeu^9^]-NKA(4–10)	-0.89 ± 0.01	7.30 ± 0.16	9.02 ± 0.39	106.7 ± 5.7		
[Arg^5^]-NKA(4–10)	-0.78 ± 0.03	8.08 ± 0.13	9.81 ± 0.38	101.8 ± 12.9		
[Lys^5^]-NKA(4–10)	-0.91 ± 0.14	7.80 ± 0.07	9.38 ± 0.46	106.9 ± 9.1		
[Arg^5^,MeLeu^9^,Nle^10^]-NKA(4-10)	-0.89 ± 0.13	6.48 ± 0.05	8.23 ± 0.32	100.4 ± 1.5		
[Arg^5^,β-Ala^8^]-NKA(4–10)	-0.86 ± 0.14	7.02 ± 0.15	8.74 ± 0.27	101.5 ± 18.4		
[Arg^5^,Nle^10^]-NKA(4–10)	-0.86 ± 0.01	6.90 ± 0.10	8.77 ± 0.34	100.8 ± 15.2		
[Lys^5^,β-Ala^8^]-NKA(4–10)	-0.85 ± 0.12	6.59 ± 0.05	8.20 ± 0.42	105.8 ± 3.4		
[Lys^5^,Nle^10^]-NKA(4–10)	-0.96 ± 0.14	6.65 ± 0.13	8.63 ± 0.34	103.7 ± 7.4		
[Arg^5^,β-Ala^8^,Nle^10^]-NKA(4–10)	-0.86 ± 0.08	5.92 ± 0.12	7.53 ± 0.41	104.9 ± 13.2		
[Lys^5^,β-Ala^8^,Nle^10^]-NKA(4–10)	-0.96 ± 0.03	5.51 ± 0.12	7.11 ± 0.50	107.0 ± 14.0		

Data are means ± SD from 2 experiments performed in duplicate (radioligand binding), 3–6 experiments performed in duplicate (intracellular Ca^2+^), or 4 experiments performed in duplicate (cAMP stimulation). For Ca^2+^ and cAMP assays, % Max refers to the maximal response observed with substance P. ND: not determined.

### Intracellular calcium response in CHO cells expressing human recombinant NK1 receptors

**Fig 10:**
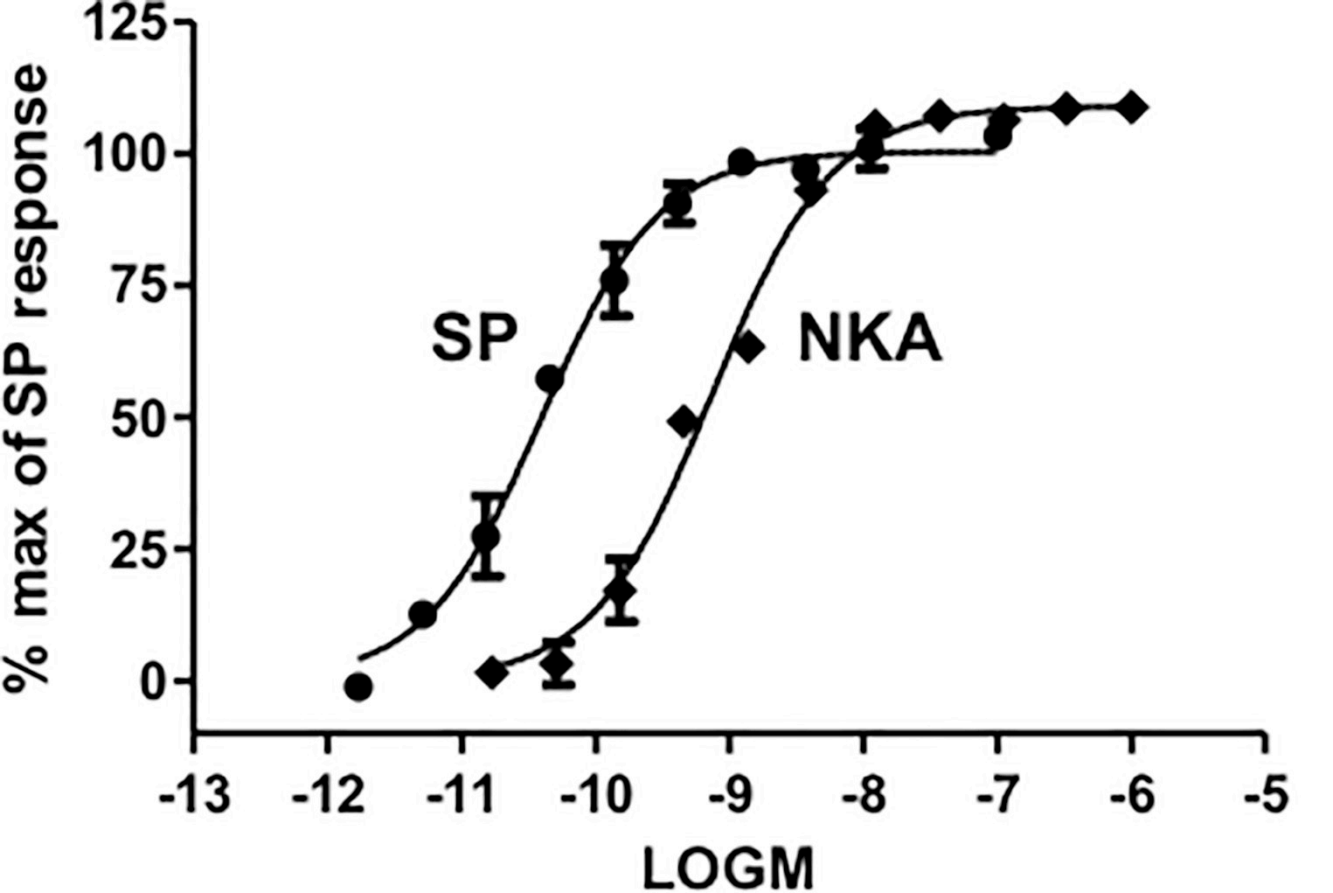
Concentration-response curves for substance P and NKA on intracellular calcium levels using human cloned NK1 receptors. Data are expressed as % of maximal response to substance P (30 nM). Each data point is the mean ± SD of data from an individual experiment performed in duplicate.

Both substance P and NKA were full agonists at NK1 receptors in the intracellular calcium assay; substance P was approximately 16-fold more potent than NKA (Fig 10; Table 3). All compounds tested elicited concentration-dependent calcium responses and were full agonists at the human NK1 receptor (Fig 4; Table 3). Substance P was the most potent agonist with a pEC_50_ of 10.48. The least potent agonist was [Lys^5^,β-Ala^8^,Nle^10^]-NKA(4–10) (pEC_50_ 7.11). Representative calcium traces are shown for NKA, substance P, [Lys^5^,MeLeu^9^,Nle^10^]-NKA(4–10) and [β-Ala^8^]-NKA(4–10) at the EC_50_ concentration in Fig 5B. All compounds tested exhibited a similar modulation of the intracellular calcium profile over time at NK1 receptors.

**Fig 11:**
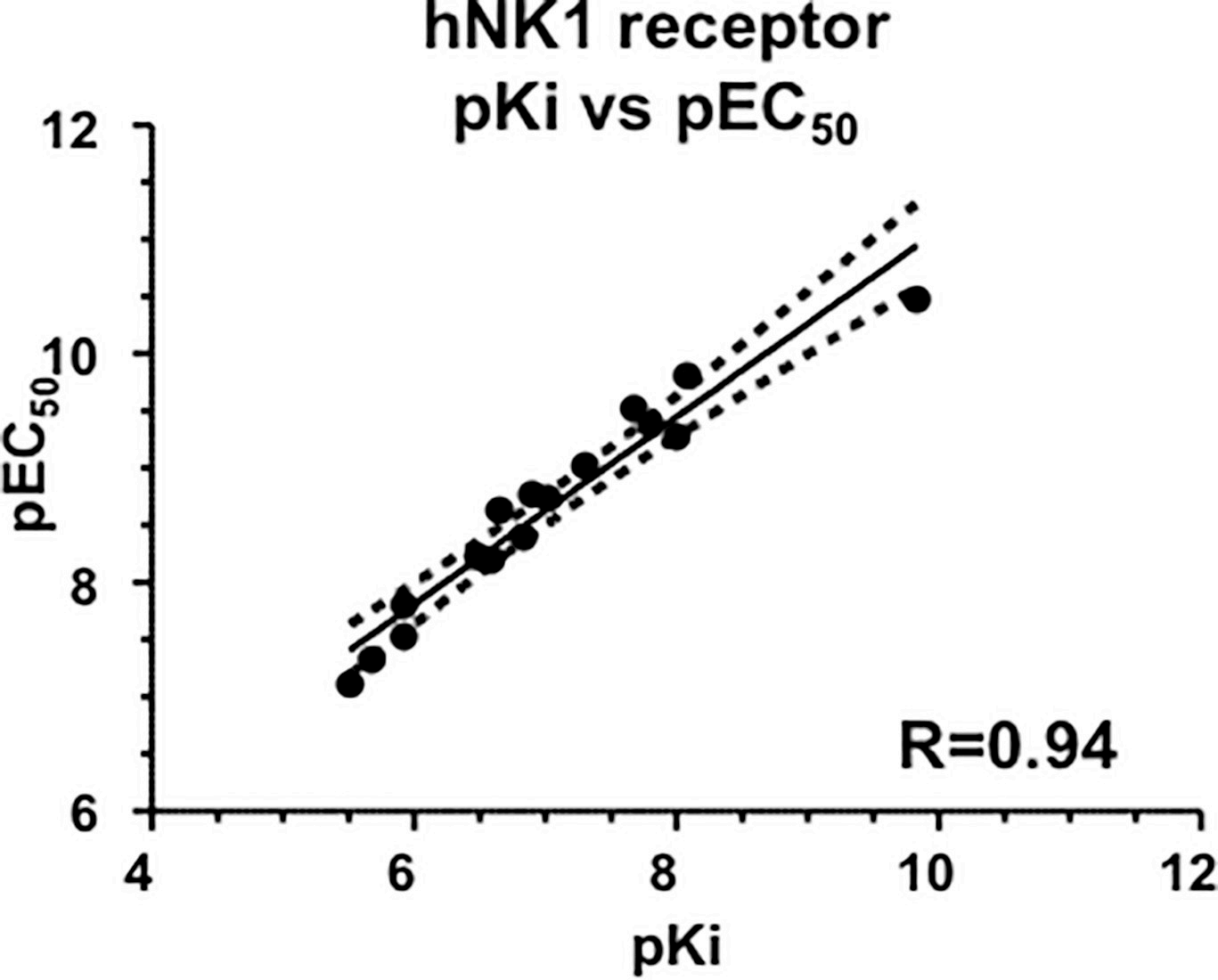
Correlation between displacement of [^3^H]-Septide binding (pKi) and intracellular calcium response (pEC_50_) at human recombinant NK1 receptors expressed in CHO cells. Scatterplot and regression analysis of pKi versus pEC_50_ values at human recombinant NK1 receptors for a series of NK2 agonists. Solid line: regression analysis; dotted lines: 95% confidence intervals.

The pKi values for displacement of [^3^H]-Septide binding and pEC_50_s for intracellular calcium response in CHO cells expressing human recombinant NK1 receptors were normally distributed (Shapiro-Wilk test, p>0.05). There was an excellent linear correlation between pKi and pEC_50_ values (R^2^ = 0.94; Fig 11).

#### Cyclic AMP Stimulation in CHO cells expressing human recombinant NK1 receptors

**Fig 12:**
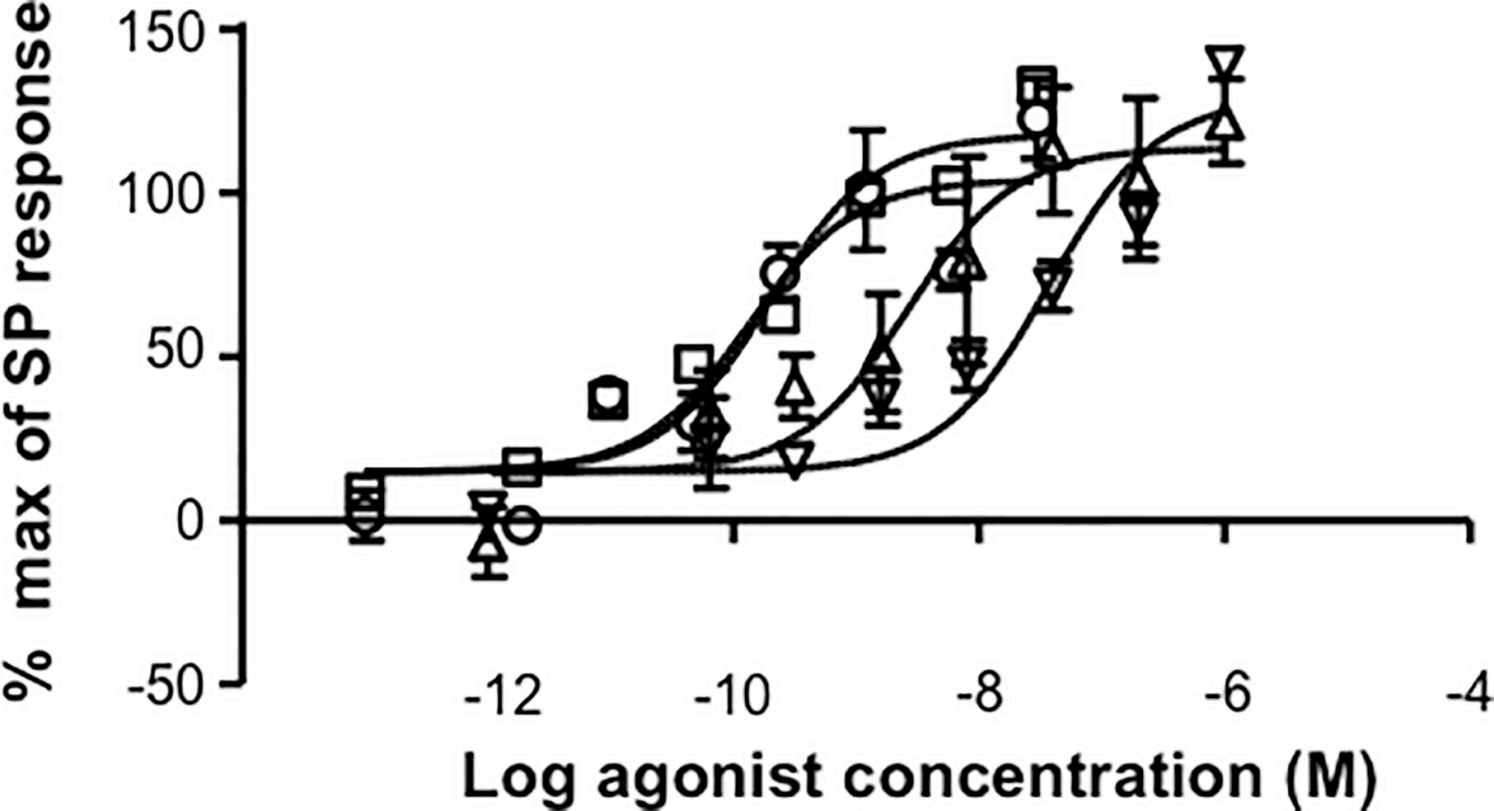
Concentration-response curves for stimulation of cyclic AMP by selected compounds using human cloned NK1 receptors. Data are expressed as % of maximal response to 30 nM substance P. Triangles are [Lys^5^,MeLeu^9^,Nle^10^]-NKA(4–10); inverted triangles are [β-Ala^8^]-NKA(4-10); squares are NKA; circles are substance P. Data are the mean ± SD of one representative experiment performed in duplicate.

NKA caused the most potent stimulation of cAMP production in CHO cells expressing NK1 receptors, followed by substance P (Fig 12; Table 3). [β-Ala^8^]-NKA(4–10) and [Lys^5^,MeLeu^9^,Nle^10^]-NKA(4–10) were notably weaker agonists than NKA and substance P. All compounds tested were full agonists.

### Selectivity for human recombinant NK2 and NK1 receptors

In order to examine whether the test compounds exhibited preferential binding to human NK2 receptors over the septide site of human NK1 receptors, the ratio of the Ki values (in nM) for displacement of [^125^I]-NKA binding to NK2 receptors and [^3^H]-Septide binding to NK1 receptors were compared. Table 4 lists the agonists in descending selectivity for displacement of [^125^I]-NKA over [^3^H]-Septide binding. [Lys^5^,MeLeu^9^,Nle^10^]-NKA(4–10) and [Arg^5^,MeLeu^9^,Nle^10^]-NKA(4–10) were more than 28-fold more selective for the human NK2 receptor over the septide site of the NK1 receptor compared with NKA.

**Table 4:** Ratio of Ki values (in nM) for displacement of [^125^I]-NKA and [^3^H]-Septide binding.

Compound	NK1 [^3^H]-Septide Ki	NK2 [^125^I]-NKA Ki	NK1/NK2 ratio
[Lys^5^,MeLeu^9^,Nle^10^]-NKA(4-10)	589	0.87	674
[Arg^5^,MeLeu^9^,Nle^10^]-NKA(4-10)	331	0.59	561
[Lys^5^,MeLeu^9^]-NKA(4–10)	50.1	0.2	251
[β-Ala^8^]-NKA(4–10)	2089	8.51	245
[Lys^5^,β-Ala^8^,Nle^10^]-NKA(4–10)	3090	16,2	191
[Arg^5^,MeLeu^9^]-NKA(4–10)	20.89	0.12	174
[Arg^5^,β-Ala^8^,Nle^10^]-NKA(4–10)	1202	6.92	174
[Lys^5^,β-Ala^8^]-NKA(4–10)	257	2.1	122
[Arg^5^,β-Ala^8^]-NKA(4–10)	95.5	0.89	107
[Lys^5^,Nle^10^]-NKA(4–10)	224	2.8	80
[Arg^5^,Nle^10^]-NKA(4–10)	126	1.74	72
NKA(4–10)	148	2.3	64
[Lys^5^]-NKA(4–10)	15,8	0.38	42
[Arg^5^]-NKA(4–10)	8.3	0.31	27
NKA	10	0.51	20

Similarly, the ratio of the EC_50_s (in nM) derived from the functional assays was determined. Table 5 lists the agonists in descending selectivity for NK2 over NK1 receptor agonist potency on calcium responses. Consistent with data obtained using radioligand binding assays, the most highly selective NK2 receptor agonists were [Lys^5^,MeLeu^9^,Nle^10^]-NKA(4–10) and [Arg^5^,MeLeu^9^,Nle^10^]-NKA(4–10), and the least selective were [Arg^5^]-NKA(4–10) and NKA. [Lys^5^,MeLeu^9^,Nle^10^]-NKA(4–10) was approximately 100-fold more selective than NKA for activation of NK2 receptors over NK1 receptors.

**Table 5:** Ratio of EC_50_s (in nM) for intracellular calcium response.

Compound	NK1 EC_50_	NK2 EC_50_	NK1/NK2 ratio
[Lys^5^,MeLeu^9^,Nle^10^]-NKA(4-10)	15.49	0.15	105
[Arg^5^,MeLeu^9^,Nle^10^]-NKA(4-10)	5.84	0.08	70
[β-Ala^8^]-NKA(4–10)	46.77	0.86	54
[Lys^5^,β-Ala^8^]-NKA(4–10)	6.31	0.18	36
[Lys^5^,β-Ala^8^,Nle^10^]-NKA(4–10)	78.22	3.00	26
[Arg^5^,β-Ala^8^,Nle^10^]-NKA(4–10)	29.51	1.16	25
[Arg^5^,β-Ala^8^]-NKA(4–10)	1.83	0.15	12
[Arg^5^,Nle^10^]-NKA(4–10)	1.71	0.20	9
[Lys^5^,MeLeu^9^]-NKA(4–10)	0.95	0.14	7
[Lys^5^,Nle^10^]-NKA(4–10)	2.36	0.38	6
[Lys^5^]-NKA(4–10)	0.41	0.09	5
NKA(4–10)	4.02	1.02	4
[Arg^5^,MeLeu^9^]-NKA(4–10)	0.30	0.16	2
NKA	0.52	0.48	1
[Arg^5^]-NKA(4–10)	0.16	0.19	0.8

In the cAMP assays, [β-Ala^8^]-NKA(4–10) was the most selective compound tested for activation of NK2 over NK1 receptors, followed by [Lys^5^,MeLeu^9^,Nle^10^]-NKA(4–10). NKA had marginal selectivity for NK2 receptors, while substance P was selective for activation of NK1 over NK2 receptors (Table 6).

**Table 6:** Ratio of EC_50_s (in nM) for cAMP stimulation.

Compound	NK1 EC_50_	NK2 EC_50_	NK1/NK2 ratio
[β-Ala^8^]-NKA(4–10)	5.62	0.023	244
[Lys^5^,MeLeu^9^,Nle^10^]-NKA(4-10)	1.78	0.024	74
NKA	0.091	0.032	2.8
Substance P	0.178	3.71	0.05

## Discussion

To our knowledge, this is the first comparison of amino acid substitutions on the affinity, potency and efficacy of peptide agonists using human recombinant NK2 and NK1 receptors. Consistent with the findings of Chassaing et al [20] and Warner et al [22] using native tissue preparations, the compound with the greatest selectivity for human recombinant NK2 versus NK1 receptors in both radioligand binding and calcium response was [Lys^5^,MeLeu^9^,Nle^10^]-NKA(4-10). As expected, an Arg^5^ substitution in combination with MeLeu^9^,Nle^10^ was also well tolerated, and this peptide was the next most selective. These two peptides with the combined MeLeu^9^ and Nle^10^ substitution were both more selective than the corresponding analogues with MeLeu^9^ or Nle^10^ (namely [Lys^5^,MeLeu^9^]-NKA(4–10), [Lys^5^,Nle^10^]-NKA(4–10), [Arg^5^,MeLeu^9^]-NKA(4–10), and [Arg^5^,Nle^10^]-NKA(4–10)). [β-Ala^8^]-NKA(4-10), which was previously characterized as the most potent and selective NK2 receptor agonist [25], had considerably lower selectivity than the Lys^5^ or Arg^5^ substitutions combined with MeLeu^9^,Nle^10^. The present studies also examined a number of novel peptides combining other substitutions that individually were known to improve NK2 receptor potency. Surprisingly, combining β-Ala^8^ with other amino acid modifications actually reduced selectivity compared with [β-Ala^8^]-NKA alone; these peptides were [Lys^5^,β-Ala^8^]-NKA(4–10), [Arg^5^,β-Ala^8^]-NKA(4–10), [Lys^5^,β-Ala^8^,Nle^10^]-NKA(4–10) and [Arg^5^,β-Ala^8^,Nle^10^]-NKA(4–10).

In the radioligand displacement assays, some Hill slopes were statistically lower than unity. The inhibition of [^125^I]-NKA binding by [Arg^5^,β-Ala^8^]-NKA(4–10) and [β-Ala^8^]-NKA(4-10) had Hill slopes of -0.70 and -0.72, respectively) and for these 2 compounds, only pIC_50_ values were calculated. Radioligand binding studies suggested that [Lys^5^,MeLeu^9^,Nle^10^]-NKA(4-10) had excellent selectivity for NK2 over NK1 receptors (674-fold) whereas for NKA, the separation was only 20-fold. Displacement of specific [^125^I]-NKA binding to human NK2 receptors or [^3^H]-Septide to human NK1 receptors was correlated with the ability of the compounds to increase intracellular calcium levels in the same cell lines. When tested at NK2 receptors, the pEC_50_ and pKi values were similar for most compounds, but for others the values differed (e.g. by 5.9-fold for [Lys^5^,MeLeu^9^,Nle^10^]-NKA(4–10) and 10-fold for [β-Ala^8^]-NKA(4–10)). Such differences between the corresponding pEC_50_ and pKi values were more pronounced for NK1 receptors, such as the 56-fold difference with [Arg^5^,MeLeu^9^,Nle^10^]-NKA(4–10) and the 74-fold difference with [Arg^5^,Nle^10^]-NKA(4–10). Such behavior may be compatible with receptor reserve and/or the existence of subpopulations of receptors with high and low affinities determined by their coupling to Gα_q/11_ proteins present in the two receptor systems employed in the study.

Ranking compounds by their relative potency to elicit calcium responses in NK2 versus NK1 receptor expressing CHO cells indicates that the most selective NK2 agonists tested were [Lys^5^,MeLeu^9^,Nle^10^]-NKA(4-10) (NK1/NK2 EC_50_ ratio = 105) and [Arg^5^,MeLeu^9^,Nle^10^]-NKA(4-10) (NK1/NK2 ratio = 70). Conversely, NKA and [Arg^5^]-NKA(4–10) were the least selective (NK1/NK2 ratio ≤ 1) and it is striking that NKA had no selectivity for NK2 over NK1 receptors in functional assays measuring calcium responses. Also noteworthy is that the selectivity of these compounds appeared to be lower for calcium responses as compared with the radioligand binding affinities. It is possible that the test compounds may have differential effects on cellular calcium dynamics in native, physiologically relevant, systems and that the recombinant cells used in our study may not be representative of this. Although the kinetics of the calcium response profile differed between NK2 and NK1 receptors (Fig 5), inspection of FLIPR traces did not reveal any difference in the profile of the calcium rise produced by the tested compounds on NK2 or NK1 receptors, suggesting that their mechanism of action was similar under these assay conditions within the same receptor subtype. The lower selectivity found in calcium response compared with binding affinities may also be related to the presence of subpopulations of receptors coupled to different second messengers. To explore this possibility, a cAMP stimulation assay, via Gs receptor coupling, was also utilized to assess the potency and maximal stimulation by the most representative NK2 receptor agonists on both NK2 and NK1 receptors. Although [Lys^5^,MeLeu^9^,Nle^10^]-NKA(4-10), [β-Ala^8^]-NKA(4–10), and NKA showed a higher potency in the cAMP stimulation assay compared with the calcium response, their selectivity for NK2 receptors was maintained across the two assays. Interestingly, [β-Ala^8^]-NKA(4–10) had the greatest selectivity (244-fold) for stimulation of cAMP via activation of NK2 over NK1 receptors, unlike its lower selectivity for calcium response (54-fold). This example of differential effects on second messenger signaling suggests the potential to develop biased NK2 receptor agonists with even greater selectivity in future.

The present studies mainly examined the increase in intracellular calcium levels in response to NK2 and NK1 receptor activation. The rank order of potency to increase calcium response or cAMP production appeared to be similar for activation of NK2 and NK1 receptors. The four compounds tested in the cAMP studies provide a foundation on which to conduct a formal correlation analysis comparing effects of a wider range of compounds on the two second messengers. Under physiological conditions, signal transduction may also involve other second messengers such as inositol phosphate and β-arrestin [26–28], and this might result in further differences in the selectivity of compounds for NK2 versus NK1 receptor activation in vivo. Additional studies are needed to characterize the signal transduction pathways activated by agonists acting at NK2 and NK1 receptors in order to determine whether functional selectivity can be further refined.

All compounds behaved as full agonists in the calcium response assays, and four selected compounds were also full agonists in cAMP assays, consistent with the high receptor reserve present in recombinant cell lines [29]. The behavior of [Lys^5^,MeLeu^9^,Nle^10^]-NKA(4-10) and [β-Ala^8^]-NKA(4–10) as full agonists at human recombinant NK2 receptors is in agreement with functional studies using isolated human colon [1, 4, 30] and bladder [5, 31, 32]. A previous study using human native NK1 receptors expressed by astrocytoma U373 cells also found a linear correlation between displacement of specific [^125^I]-NKA binding (to the septide site) and stimulation of inositol phosphate by a range of compounds that included 4 of those examined in the present study (NKA, [Lys^5^,MeLeu^9^,Nle^10^]-NKA(4-10), [β-Ala^8^]NKA(4–10) and [Lys^5^]NKA(4-10); [15]). Functional assays using tissues from other species (rabbit pulmonary artery for NK2 and dog carotid artery for NK1) also found a similar rank order of selectivity for NK2 over NK1 receptors of [β-Ala^8^]NKA(4-10) >> NKA(4–10) > NKA [25]. Thus, there is good agreement between the findings from the present study using human recombinant receptors and published studies using preparations of human colon, bladder and astrocytoma cells, as well as functional assays from nonhuman species, regarding the relative affinity and efficacy of the agonists tested.
